# Acquisition of Daptomycin Resistance by *Enterococcus faecium* Confers Collateral Sensitivity to Glycopeptides

**DOI:** 10.3389/fmicb.2022.815600

**Published:** 2022-04-12

**Authors:** Weiliang Zeng, Luozhu Feng, Changrui Qian, Tao Chen, Sipei Wang, Ying Zhang, Xiangkuo Zheng, Lingbo Wang, Shixing Liu, Tieli Zhou, Yao Sun

**Affiliations:** ^1^Key Laboratory of Clinical Laboratory Diagnosis and Translational Research of Zhejiang Province, Department of Clinical Laboratory, The First Affiliated Hospital of Wenzhou Medical University, Wenzhou, China; ^2^School of Laboratory Medicine and Life Science, Wenzhou Medical University, Wenzhou, China; ^3^School of Basic Medical Sciences, Wenzhou Medical University, Wenzhou, China

**Keywords:** collateral sensitivity, resistance mechanisms, *Enterococcus faecium*, daptomycin, glycopeptides, fitness cost

## Abstract

Daptomycin is a last-line antibiotic used in the treatment of multidrug-resistant *Enterococcus faecium* infections. Alarmingly, daptomycin-resistant *E. faecium* isolates have emerged. In this study, we investigated the evolution and mechanisms of daptomycin resistance in clinical *E. faecium* isolates and the corresponding acquisition of collateral sensitivity (CS) as an evolutionary trade-off. We evolved daptomycin resistance in six daptomycin-susceptible *E. faecium* isolates to obtain daptomycin-resistant mutants. The six *E. faecium* strains successfully acquired high-level resistance to daptomycin *in vitro*, but this led to fitness costs in terms of growth, *in vitro* competition, and virulence. Mutations in *liaFSR*, *yycFG*, and *cls*; increased surface positive charge; thicker cell walls; and elevated expression of *dltABCD* and *tagGH* were observed in daptomycin-resistant mutants. Surprisingly, we observed the emergence of CS in SC1762 isolates after the induction of daptomycin resistance. Compared with parental strains, the SC1174-D strain (i.e., daptomycin-resistant mutant of SC1174; non-CS) showed significantly upregulated expression of the *vanA* gene cluster. However, in SC1762-D (i.e., daptomycin-resistant mutant of SC1762), all *vanA* cluster genes except the *vanX* gene were obviously downregulated. Further *in silico* analyses revealed that an IS*1216E-*based composite transposon was generated in SC1762-D, and it disrupted the *vanH* gene, likely affecting the structure and expression of the *vanA* gene cluster and resulting in resensitization to glycopeptides. Overall, this study reports a novel form of CS between daptomycin and glycopeptides in *E. faecium*. Further, it provides a valuable foundation for developing effective regimens and sequential combinations of daptomycin and glycopeptides against *E. faecium*.

## Introduction

*Enterococcus faecium* is a ubiquitous and opportunistic pathogen. It is a member of the *ESKAPE* (*E. faecium*, *Staphylococcus aureus*, *Klebsiella pneumoniae*, *Acinetobacter baumannii*, *Pseudomonas aeruginosa*, and *Enterobacter* species) family of pathogens, which are well-known for causing frequent and hard-to-treat healthcare-associated infections (Pendleton et al., 2013; Mulani et al., 2019). *E. faecium* can cause various infections in hospital and community settings, ranging from urinary tract infections (UTIs) to severe life-threatening infections such as bacteremia (Contreras et al., 2019). It has been reported that *E. faecium* infections are becoming increasingly common (Li et al., 2017). Vancomycin and teicoplanin were thought to be the most effective glycopeptides for treating *Enterococcal* infections. However, since vancomycin-resistant *E. faecium* (VRE*fm*) was first identified in England and France in 1986 (O’Driscoll and Crank, 2015), such drug-resistant strains have been encountered worldwide and now represent a great public health threat due to their high mortality rates (Arias and Murray, 2012).

In 2003, daptomycin (DAP)—a cyclic lipopeptide antibacterial agent—was approved by the Food and Drug Administration for the treatment of complex skin and soft tissue infections, due to its rage of activity against methicillin-resistant *S. aureus* (MRSA) and vancomycin-resistant *Enterococci* (VRE) (Frankenfeld et al., 2018). However, with its extensive use in clinical settings, the number of treatment failures reported with DAP has been increasing (Jones et al., 2008; Lellek et al., 2015; Ji et al., 2020).

The mechanisms underlying DAP resistance in both *S*. *aureus* and *Enterococci* have been described. Studies have shown that gene mutations are responsible for DAP resistance. These include mutations in genes encoding cell-envelope homeostasis proteins, *liaFSR* (two-component regulatory system encoding genes), *yycFG* (i.e., three-component regulatory system encoding genes), and genes encoding enzymes associated with cell membrane phospholipid metabolism, *cls* (cardiolipin synthase), *cfa* (cyclopropane fatty acid synthetase), and *mprF* (multiple peptide resistance factor) (Munita et al., 2012; Werth et al., 2014; Lellek et al., 2015; Bayer et al., 2016; Prater et al., 2019; Ji et al., 2020). Changes in surface charge, membrane depolarization, and cell-wall thickness also contribute to DAP resistance (Jones et al., 2008; Arias et al., 2011; Miller et al., 2016; Kang et al., 2017). Although the mechanisms underlying DAP resistance are diverse, the dynamic evolution of acquired DAP resistance and the related mechanisms remain to be fully elucidated.

Under antimicrobial selection pressure, bacteria often make adaptive changes depending on the antibiotic environment. These changes also represent the evolutionary trade-off that bacteria make to acquire antibiotic resistance. It has been demonstrated that the acquisition of resistance to a specific antimicrobial can simultaneously increase or decrease a bacterium’s level of resistance to another antimicrobial. This process is called cross-resistance (CR) or collateral sensitivity (CS) (Roemhild et al., 2020). Notably, researchers have suggested that CS can be exploited to slow down or even reverse antibiotic resistance (Imamovic et al., 2018; Lázár et al., 2018). Hence, it is necessary to fully understand the mechanisms of CS, especially for a last-line-of-defense drug such as DAP. Nevertheless, there are few reports on CS for DAP. Canfield et al. (2021) reported that the sensitization of *E. faecium* to antimicrobials that act on the cell wall (e.g., ceftriaxone and ampicillin) and membrane (e.g., DAP) after the acquisition of phage resistance in *E. faecium* infected with lytic bacteriophages. Moreover, DAP can reverse rifampicin resistance in some strains of VRE through mechanisms that are currently unclear (Rand et al., 2007). Interestingly, we also observed the emergence of CS between DAP and glycopeptides in *E. faecium* after exposure to DAP. The aim of this study was to explore the evolution of DAP resistance and the corresponding CS in *E. faecium* with the aim of providing a basis for formulating more reasonable clinical treatment strategies and promoting the development of new antimicrobial combinations based on CS.

## Materials and Methods

### Bacterial Strains and Media

This study used strains obtained from the First Affiliated Hospital of Wenzhou Medical University. The Institutional Ethics Committee of the First Affiliated Hospital of Wenzhou Medical University exempted the study from ethics review and approval because the study was observational in nature and mainly focused on bacteria, with no patient-related interventions.

We collected and identified six DAP-susceptible (DAP-S) strains (i.e., SC1174, SC1379, SC1762, SC1543, SC1706, and SC1726), which were isolated between 2017 and 2018 from the First Affiliated Hospital of Wenzhou Medical University. These isolates were used as parental strains to generate DAP-resistant (DAP-R) mutants *in vitro*. Considering that calcium is necessary for the antimicrobial activity of DAP, both calcium (50 μg/ml) and magnesium ions (12.5 μg/ml) were added to Mueller-Hinton broth and Luria-Bertani (LB) broth in this study.

### Adaptive Laboratory Evolution of *Enterococcus faecium*

To examine the evolution of DAP resistance in *E. faecium*, an adaptive laboratory evolution experiment *in vitro* was performed in six parental strains, with slight modifications, and DAP-R mutants were obtained (Bhardwaj et al., 2018). The experimental design of the evolution experiment was shown in Supplementary Figure 1. Specifically, a single pure colony of the DAP-S parental strains was randomly picked, inoculated in 3 ml fresh LB broth, and allowed to grow to the logarithmic phase. Then, 30 μl of the overnight culture was transferred to 2.97 ml fresh LB broth containing graded concentrations of DAP: 1/2 × minimal inhibitory concentration (MIC), 1 × MIC, 2 × MIC, and 4 × MIC. Subsequently, the cultures were incubated at 37°C overnight without shaking. The tube with visible growth at the highest DAP concentration was used as the inoculum, which was cultured for 3 days in fresh LB broth with the DAP concentration, and then the inoculum was exposed to a higher concentration. The stability of the DAP-R mutants was confirmed using serial cultures in fresh LB broth without DAP (Li et al., 2017). The continuous passage of the parental strains in LB broth without DAP served as the control for this set of experiments.

### Antimicrobial Susceptibility Testing

According to the guidelines of the Clinical and Laboratory Standards Institute [CLSI] (2020), the MIC of DAP and commonly used antibiotics was determined for DAP-S and DAP-R strains using the broth microdilution and agar dilution methods. *E. faecalis* ATCC 29212 was used as the control strain. The results were also interpreted based on CLSI standards. The experiment was repeated in triplicate.

### Polymerase Chain Reaction Amplification and DNA Sequencing

Genomic DNA was extracted from the experimental strains using a Biospin bacterial genome DNA extraction kit (Shanghai Boyun Biotech Co., Ltd. Shanghai, China.) and used as the template in polymerase chain reaction (PCR). DAP-resistance genes (i.e., *liaFSR*, *yycFG*, *cls*, and *cfa*) and glycopeptide-resistance gene (i.e., *vanRSHAXYZ*) were amplified using specific primers (Supplementary Table 1; Munita et al., 2012; Werth et al., 2014; Lellek et al., 2015). The PCR products were sequenced. In addition to the genome of the parental strains, nucleotide sequences were also compared with the genome of the standard strains *E. faecium* DO (accession number: CP003583) and *E. faecium* BM4147 (accession number: M97297) using BLAST^1^ (Qin et al., 2012; Cha et al., 2013).

### Cytochrome C Binding Assay

The cytochrome C binding assay was performed using the parental strains and their mutants (Kang et al., 2017). Briefly, bacterial suspensions were allowed to regrow to the logarithmic phase after overnight culture. Then, cells were washed twice with 3-(N-Morpholino) propanesulfonic acid sodium salt (MOPS, *pH* = 7.0) after centrifugation, and the bacterial suspension was adjusted to an *OD*_600_ of 1.0. Cytochrome C was prepared in MOPS (5 mg/ml, *pH* = 7.0), and then, 100 μl of this solution was added to 900 μl of the bacterial suspension. After 30 min of incubation at room temperature, samples were centrifuged at 12,000 *g* for 5 min. The *OD*_530_ was measured spectrophotometrically. Lower levels of free cytochrome C in the supernatant indicated a greater net positive charge on the bacterial surface. The experiment was repeated at least three times.

### Transmission Electron Microscopy

Two parental strains (SC1174 and SC1762) and their evolved strains (SC1174-D and SC1762-D) were selected to measure the cell-wall thickness using transmission electron microscopy (TEM), as described previously (Arias et al., 2011). After overnight culture in LB broth, 1 ml of the bacterial suspension was pelleted and washed three times using 0.1 M Millonig’s phosphate buffer (*pH* = 7.0). The pellet was then resuspended in 1 ml glutaraldehyde and further processed in the Electron Microscope Room of Wenzhou Medical University. For each tested strain, the cell-wall thickness of at least 25 cells was measured at 40,000× and 60,000× magnification. Mean differences were compared using the Student’s *t*-test, with *P*-values of <0.05 indicating statistical significance.

### Efflux Pump Inhibition Testing

The efflux pump inhibition test was performed to identify the relationship between efflux pumps and CS. The tested efflux pump inhibitors were as follows: carbonyl cyanide m-chlorophenylhydrazone (CCCP, 6 μg/ml), Phe-Arg-β-naphthylamide (PAβN) (20 μg/ml), omeprazole (OME, 100 μg/ml), reserpine (RES, 20 μg/ml), and chlorpromazine (CHL, 20 μg/ml). The MICs of *E. faecium* with or without the efflux pump inhibitors were compared to measure the efflux activities. Based on the literature, a positive phenotype for a particular strain was indicated by an MIC decrease of ≥4 after the supplementation of the efflux pump inhibitor (vs. without the efflux pump inhibitor) (Lin et al., 2020).

### Quantitative Real-Time Polymerase Chain Reaction

Total RNA was extracted with an RNeasy Mini Kit (QIAGEN, Valencia, CA, United States). Using a cDNA synthesis kit (Takara, Shiga, Japan), purified RNA was reverse transcribed to cDNA, which was used as a template for quantitative real-time PCR (qRT-PCR). The expression levels of *dltABCD*, *tagGH*, and *vanRSHAXYZ* genes were detected using their respective specific primers (Supplementary Table 1). The housekeeping gene *16S rRNA* was used as the corresponding internal control, and transcript levels were calculated using the 2^–Δ^ ^Δ^ *^Ct^* method. At least two independent runs were performed for each cDNA sample.

### Whole-Genome Sequencing and Bioinformatics Analysis

The genomic DNA of SC1762 and SC1762-D was extracted using an AxyPrep bacterial genomic DNA miniprep kit (Axygen Scientific, Union City, CA, United States). Whole-genome sequencing (WGS) was performed using a standard run on the Illumina HiSeq 2500 and Pacific Bioscience (PacBio) systems by the Shanghai Personal Biotechnology Co., Ltd. (Shanghai, China). Complete genomes were assembled by Canu (Koren et al., 2017) using long reads and then improved by Pilon (Walker et al., 2014) using short reads. Genomic sequences were annotated using Prokka (Seemann, 2014) and corrected using BLAST searches against the UniProtKB/Swiss-Prot, RefSeq, ISfinder (Siguier et al., 2006), and CARD (Jia et al., 2017) databases. Gene organization diagrams were generated using the Python script and modified with Inkscape^2^. The raw data from WGS were submitted and are accessible under NCBI accession numbers CP085894-CP085905 (SC1762) and CP085906-CP085917 (SC1762-D).

### Measurement of Growth Kinetics

The growth rate of DAP-R mutants and the parental strains were investigated (Li et al., 2017). After growth in LB broth at 37°C for 24 h, cultures of these strains were diluted to an *OD*_600_ of 0.01 and incubated at 37°C for 24 h with agitation at 200 rpm. Growth curves were generated by plotting the changes in the *OD*_600_ value over time. The experiment was repeated in triplicate, and the average values were used for estimating growth parameters.

### *In vitro* Competition Experiments

*In vitro* competition experiments were performed in triplicate to measure the fitness cost between parental strains and their mutants (Li et al., 2017). Cultures of exponentially growing cells (both parental strains and mutant strains) were adjusted to a dilution of 1 × 10^3^ colony forming unit (CFU)/ml, and then equal volumes of the parental and mutant strains were combined. Subsequently, 1 ml of the mixture was added to 19 ml LB broth and cultured at 37°C with agitation at 200 rpm for 24 h, which corresponds to approximately 20 cell generations. Serial 10-fold dilutions were plated in duplicate on DAP-free LB agar and LB agar containing 4 μg/ml DAP (this concentration inhibited the growth of all parental strains). After overnight incubation at 37°C for 24 h, the CFUs of the DAP-R colonies and parental strains were counted. The competition index (CI) indicates the adaptive difference and was defined as the ratio between the number of CFUs from the parental strains and that from the mutants. The *CI* values were calculated for each independent competition assay, and the median values were obtained.

### Crystal Violet Biofilm Assays

Quantifications of biofilm-bound crystal violet were performed for parental strains and evolved DAP-R mutants as previously reported, with slight modifications (Jung et al., 2019). For the biofilm formation assays, isolates were incubated at 37°C with shaking at 200 rpm overnight. Then, culture suspensions were diluted in fresh LB broth (1:100) and inoculated into a 96-well polystyrene microtiter plate (200 μl per well), which was incubated at 37°C for 48 h. After incubation, planktonic cells were removed, and 200 μl of 0.1% crystal violet was added to each well. The plate was incubated for 20 min at 37°C before it was washed twice with sterile water. Finally, 95% ethanol was used to dissolve the stained biofilm, and the absorbance at *OD*_600_ was measured for quantification. Untreated wells supplemented with sterile LB broth served as the control. All experiments were conducted in triplicate.

### *Galleria mellonella* Infection Assays

*Galleria mellonella* larvae were used as infection models to compare the virulence of parental strains and evolved DAP-R mutants; a slightly modified version of a previously described method was used (Taglialegna et al., 2019). Twelve caterpillars weighing 200–250 mg were randomly selected for testing each isolate. Then, 10 μl of bacterial suspension containing 10^8^ CFU/ml or normal saline (NS) was injected into the last left proleg of the *G. mellonella* larvae using a 25 μl Hamilton precision syringe. Worms were incubated at 37°C in the dark, and their survival rates were observed and recorded every 24 h over a period of 6 days. The survival rates were assessed using the Kaplan–Meier analysis and the log-rank test. All experiments were performed in three independent replicates.

### Statistical Analysis

The paired Student’s *t*-test was performed using SPSS software version 26.0. The statistical analysis of growth rate was performed using the GraphPad Prism 8.0 software and one-way analysis of variance (ANOVA), and the log-rank test was used to analyze the survival rates of *G. mellonella larvae* infection models. *P*-values of < 0.05 were considered significant.

## Results

### Evolved Daptomycin-Resistant *Enterococcus faecium* Mutants

**FIGURE 1:**
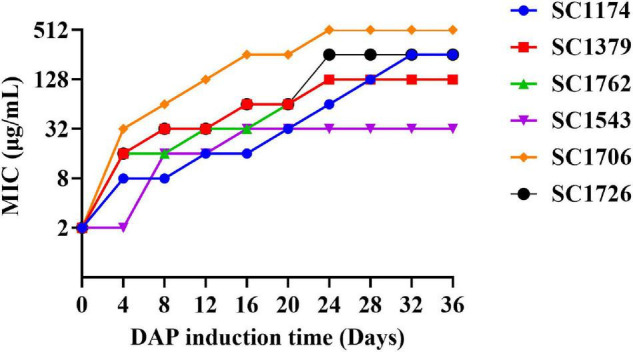
*Enterococcus faecium* adapts to daptomycin. The *in vitro* evolution of reduced daptomycin (DAP) susceptibility in evolved DAP-resistant (DAP-R) *E. faecium* through serial passages in increasing concentrations of DAP for a period of 36 days.

Daptomycin-resistant mutants were generated successfully through the adaptive laboratory evolution. The evolved DAP resistance remained stable after serial passages of *E. faecium* in a DAP-free growth medium. As shown in Figure 1, the DAP resistance of *E. faecium* increased with an increase in the induction concentration of DAP and the induction time. On the fourth day of DAP induction, DAP-R mutants were screened. After a 36-day induction period, the six mutants exhibited a 16–256× increase in the DAP MIC compared with their wild-type counterparts (Table 1). In summary, the DAP-R *E. faecium* mutants could be rapidly selected through serial passaging across a sub-lethal gradient of DAP.

**TABLE 1:** Activity of daptomycin and other antibiotics against parental and mutant strains of *Enterococcus faecium.*

Isolates	MIC (μ g/mL)										
	DAP	AMP	CIP	LVX	NIT	PEN	ERY	LNZ	TCY	VAN	TEC
SC1174	2*^S^*	512*^R^*	512*^R^*	128*^R^*	128*^R^*	512*^R^*	0.25*^S^*	2*^S^*	0.5*^S^*	512*^R^*	128*^R^*
SC1174-D	256*^R^*	512*^R^*	512*^R^*	128*^R^*	128*^R^*	512*^R^*	0.25*^S^*	2*^S^*	0.5*^S^*	512*^R^*	128*^R^*
SC1379	2*^S^*	512*^R^*	512*^R^*	128*^R^*	128*^R^*	512*^R^*	512*^R^*	1*^S^*	>16*^R^*	512*^R^*	256*^R^*
SC1379-D	128*^R^*	512*^R^*	512*^R^*	128*^R^*	128*^R^*	512*^R^*	512*^R^*	1*^S^*	>16*^R^*	512*^R^*	256*^R^*
SC1762	2*^S^*	512*^R^*	256*^R^*	128*^R^*	128*^R^*	512*^R^*	512*^R^*	1*^S^*	0.25*^S^*	512*^R^*	128*^R^*
SC1762-D	128*^R^*	512*^R^*	256*^R^*	128*^R^*	128*^R^*	512*^R^*	512*^R^*	1*^S^*	0.25*^S^*	2*^S^*	≤2*^S^*
SC1543	2*^S^*	8*^S^*	1*^S^*	2*^S^*	128*^R^*	16*^R^*	512*^R^*	1*^S^*	0.5*^S^*	1*^S^*	≤2*^S^*
SC1543-D	32*^R^*	8*^S^*	1*^S^*	2*^S^*	128*^R^*	16*^R^*	512*^R^*	1*^S^*	0.5*^S^*	1*^S^*	≤2*^S^*
SC1706	2*^S^*	2*^S^*	4*^R^*	8*^R^*	128*^R^*	4*^S^*	8*^R^*	2*^S^*	0.25*^S^*	1*^S^*	≤2*^S^*
SC1706-D	512*^R^*	2*^S^*	8*^R^*	8*^R^*	128*^R^*	4*^S^*	8*^R^*	2*^S^*	0.25*^S^*	1*^S^*	≤2*^S^*
SC1726	2*^S^*	2*^S^*	8*^R^*	8*^R^*	256*^R^*	8*^S^*	8*^R^*	2*^S^*	0.25*^S^*	1*^S^*	≤2*^S^*
SC1726-D	256*^R^*	2*^S^*	8*^R^*	8*^R^*	256*^R^*	8*^S^*	8*^R^*	2*^S^*	0.25*^S^*	1*^S^*	≤2*^S^*

*MIC, minimum inhibitory concentration; DAP, daptomycin; AMP, ampicillin; CIP, ciprofloxacin; LVX, levofloxacin; NIT, nitrofurantoin; PEN, penicillin; ERY, erythromycin; LNZ, linezolid; TCY, tetracycline; VAN, vancomycin; TEC, teicoplanin. -D, evolved DAP-R mutant; S, susceptibility; R, resistance.*

### Emergence of Collateral Sensitivity in Daptomycin-Resistant *Enterococcus f*aecium** Mutants

To determine whether the bacterial resistance phenotype can change after DAP induction, we first determined the susceptibility of the mutant strains to clinically relevant antibiotics (Table 1). Compared with the parental strain SC1762, the mutant strain SC1762-D exhibited a 64× increase in the DAP MIC, becoming clinically resistant to DAP. Interestingly, it also exhibited a 256× and 64× decrease in the MIC of vancomycin and teicoplanin, becoming clinically sensitive to these antibiotics, indicating CS between DAP and glycopeptides in *E. faecium* SC1762 and SC1762-D. Except for SC1762 and SC1762-D, as shown in Table 1, the antibiotic resistance phenotype of the evolved DAP-R strains was consistent with that of the corresponding parental strains.

### Mutations in *liaFSR*, *yycFG*, and *cls* Were Related to Daptomycin Resistance

**FIGURE 2:**
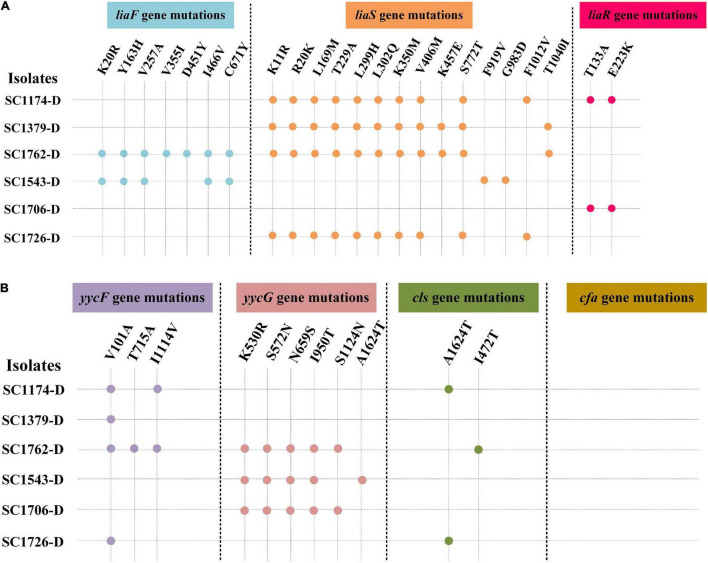
Genes mutations in daptomycin-resistant mutants. DAP resistance gene mutations in the evolved DAP-R mutants. -D, evolved DAP-R isolates. **(A)** *liaFSR* gene mutations; **(B)** *yycFG*, *cls*, and *cfa* gene mutations. Blue, orange, red, purple, pink, and green dots represent the mutation sites in the *liaF*, *liaS*, *liaR*, *yycF*, *yycG*, and *cls* genes, respectively.

We evaluated the mechanisms of DAP resistance in the evolved mutants. Results of PCR amplification showed that different strains had different gene mutation patterns (Figure 2 and Supplementary Figure 2). Mutations were identified in *liaFSR*, *yycFG*, and *cls* genes (except *cfa*), and the mutation sites were not exactly the same in different DAP-R mutants. Further, the mutation frequency for the same gene was also different in each mutant.

### Increased Cell Surface Charge and Cell-Wall Thickness in Daptomycin-Resistant Mutants

**FIGURE 3:**
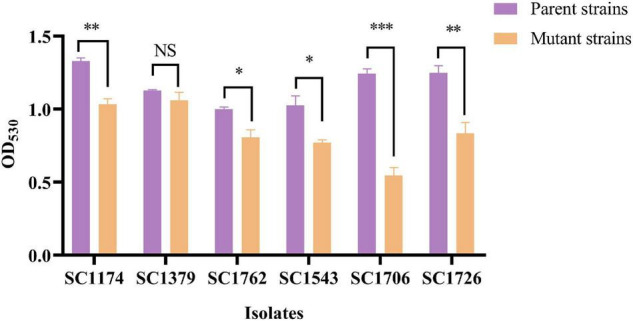
Relative positive surface charge examined based on cytochrome C binding. Evolved DAP-R mutants, except SC1379-D, had less free cytochrome C in the supernatant than their parental strains, indicating that they carried more net positive charge. Data are presented as mean values with error bars indicating standard deviations of the means of results from duplicate experiments, and *P-*values were calculated by the paired Student’s *t*-test (*P < 0.05; **P < 0.01; and ***P < 0.001; NS means no significant difference).

**FIGURE 4:**
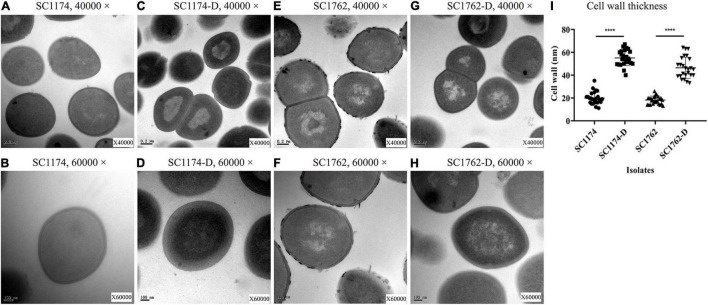
Cell-wall thickness examined using transmission electron microscopy. **(A)** SC1174, 40,000×; **(B)** SC1174, 60,000×; **(C)** SC1174-D, 40,000×; **(D)** SC1174-D, 60,000×; **(E)** SC1762, 40,000×; **(F)** SC1762, 60,000×; **(G)** SC1762-D, 40,000×; **(H)** SC1762-D, 60,000×; and **(I)** the cell-wall thickness of at least 25 cells was measured for SC1174, SC1174-D, SC1762, and SC1762-D. Data were presented as the mean and standard deviation. Asterisks indicate statistical significance using the paired Student’s *t*-test (****P < 0.0001).

Cytochrome C binding assays revealed that all DAP-R mutants, except for SC1379-D, had significantly higher positive charge on their surface than the corresponding parental strains (Figure 3). TEM data showed that the cell-wall thickness of SC1174-D and SC1762-D was greater than that of SC1174 (average thickness, 55.09 ± 6.55 nm vs. 19.97 ± 5.26 nm; *P* < 0.0001) and SC1762 (average thickness, 46.456 ± 9.08 nm vs. 18.21 ± 3.36 nm; *P* < 0.0001) (Figure 4), respectively. Collectively, these results suggested that the evolution of DAP resistance *in vitro* in *E. faecium* was associated with genetic mutations, changes in the positive charge on the cell surface, and the thickness of the cell wall.

### Expression Levels of *dltABCD* and *tagGH*

**FIGURE 5:**
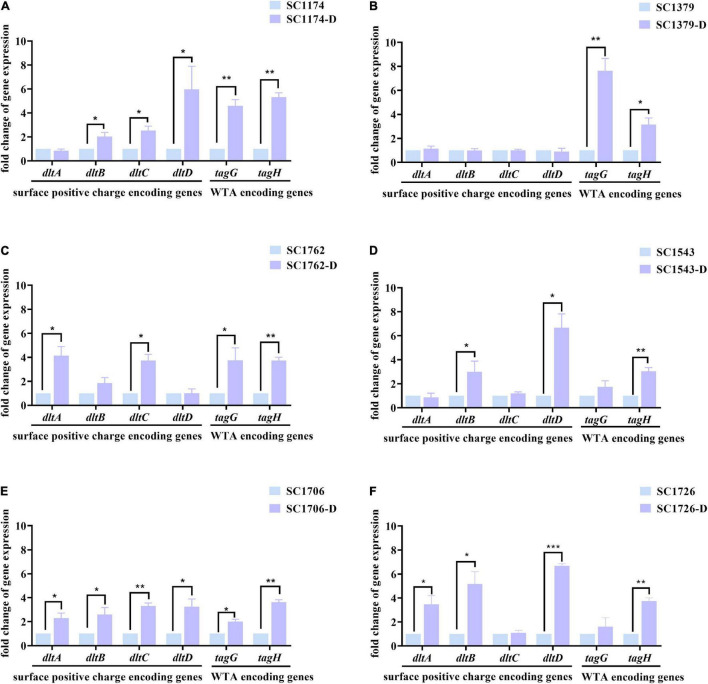
Relative expression levels of *dltABCD* and *tagGH* in the parental strains and DAP-R mutants. Values were normalized based on the internal control gene, *16S rRNA*; data from the parental strain were normalized to 1 to allow the comparison of data across different samples. **(A–F)** show the expression levels of the surface positive charge-encoding genes in *dltABCD* and the cell-wall teichoic acid (WTA)-encoding genes in *tagGH* in SC1174, SC1379, SC1762, SC1543, SC1706, SC1726, and their corresponding evolved DAP-R mutants, respectively. Data represent the mean values from three independent experiments with error bars indicating standard deviations, and asterisks denote the significance of differences in expression by the paired Student’s *t*-test (*P < 0.05; **P < 0.01; and ***P < 0.001).

We also determined the expression levels of surface positive charge-encoding genes *dltABCD* and cell-wall teichoic acid (WTA)-encoding genes *tagGH* in the parental strains and their mutants. Compared with the parental strains, all mutants except SC1379-D showed higher expression levels of *dltABCD* (Figure 5). Elevated *tagGH* expression was also observed among the mutants (Figure 5). These changes in gene expression could explain the increase in the positive surface charge and cell-wall thickness.

### Phenotypic Detection of Efflux Pump Activity

In order to explore the potential mechanisms of CS, the parental strain SC1762 and the DAP-R mutant SC1762-D (with CS) were used as the experimental group (CS group). Simultaneously, the parental strain SC1174 and DAP-R mutant SC1174-D (without CS) were randomly selected as the control group (non-CS group).

We first evaluated the mechanism of CS between DAP and glycopeptides using efflux pump inhibitors. We found that the DAP MIC of the four tested strains decreased by ≥fourfold after exposure to the efflux inhibitors CCCP and CHL; SC1174-D and SC1762-D had a positive efflux pump phenotype relative to their wild-type strains when an OME combination was used. Similarly, the MIC of vancomycin and teicoplanin also reduced by ≥fourfold among the four tested strains after exposure to CCCP. The efflux pump phenotype of both SC1174-D and SC1762-D was positive after the addition of PAβN, OME, RES, and CHL (Table 2). Taken together, these data suggested that there was no difference in the efflux pump phenotypes for DAP, teicoplanin, and vancomycin between the CS group and the non-CS group.

**TABLE 2:** Efflux pump phenotype testing.

Isolates	SC1174	SC1174-D	SC1762	SC1762-D
	
	MIC (μ g/mL)			
DAP	2	256	2	128
DAP + CCCP	**0.25**	**32**	**0.5**	**8**
DAP + PAβN	2	128	2	128
DAP + OME	2	**32**	1	**32**
DAP + RES	2	256	2	128
DAP + CHL	**0.25**	**2**	**0.25**	**1**
VAN	512	512	512	2
VAN + CCCP	**128**	**64**	**64**	**<0.5**
VAN + PAβN	512	**16**	512	**<0.5**
VAN + OME	512	**1**	512	**<0.5**
VAN + RES	512	**32**	512	**<0.5**
VAN + CHL	512	**32**	512	**<0.5**
TEC	128	128	128	≤2
TEC + CCCP	**32**	**32**	**16**	**<0.25**
TEC + PAβN	64	**32**	128	**<0.25**
TEC + OME	128	**16**	128	**<0.25**
TEC + RES	128	**32**	128	**<0.25**
TEC + CHL	128	**16**	128	**<0.25**

*DAP, daptomycin; VAN, vancomycin; TEC, teicoplanin; CCCP, carbonyl cyanide 3-chlorophenylhydrazone; PAβN, Phe-Arg-β-naphthylamide; OME, omeprazole; RES, reserpine; CHL, chlorpromazine; MIC, minimum inhibitory concentration; -D, evolved DAP-R strains. Bold data represent that the MIC value of DAP, VAN, or TEC decreased ≥fourfold after the supplementation of the efflux pump inhibitors (vs. without the efflux pump inhibitors).*

### Mutations and Expression Levels of the *vanA* Gene Cluster

**FIGURE 6:**
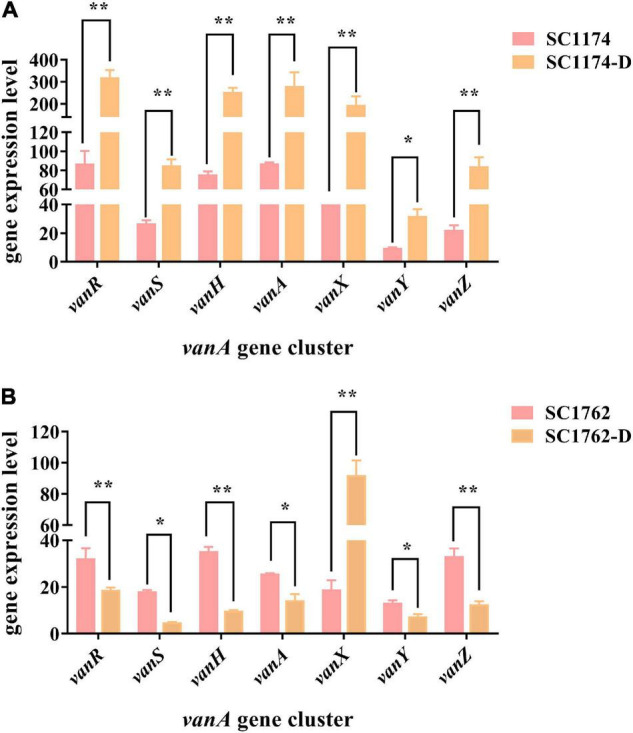
Relative expression levels of the *vanA* gene cluster in four *E. faecium* isolates. Expression levels of the *vanA* gene cluster in the non-CS group **(A)** and the CS group **(B)**. Data represent the mean values from three independent experiments with error bars indicating standard deviations, and asterisks denote the significance of differences in expression by the paired Student’s *t*-test (*P < 0.05 and **P < 0.01).

Genetic alterations and the relative expression levels of the *vanA* gene cluster were compared among the parental strains and DAP-R mutants using PCR and qRT-PCR. After comparisons with the standard strain *E. faecium* BM4147 and the corresponding parental strain, deletions or mutations in the *vanA* gene cluster were not detected in the CS and non-CS groups (data not shown). However, qRT-PCR data indicated that the expression of the *vanA* gene cluster was significantly higher in SC1174-D (Figure 6A) observably lower in SC1762-D (excluding *vanX*) than in the corresponding parental strains (Figure 6B).

### Whole-Genome Sequencing Analysis

**FIGURE 7:**
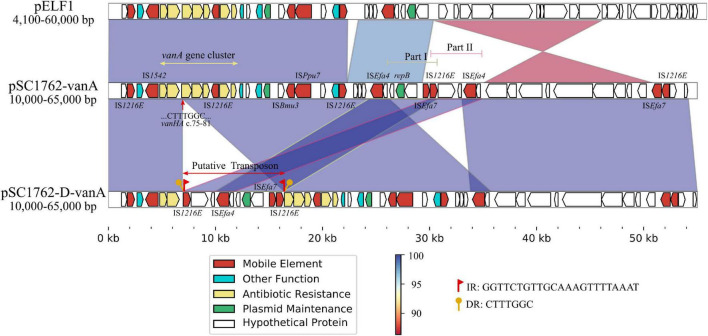
Comparative genetic structures and the genetic environment of the *vanA* gene cluster. The arrows represent sequence units or genes and are colored based on gene functional classification. Orthologous regions are connected and color coded.

Whole-genome sequencing was performed on SC1762 and SC1762-D to explain the reduced expression of the *vanA* gene cluster. Genomic features are shown in Supplementary Table 2. The plasmid (pSC1762-*vanA*) harboring the *vanA* gene cluster was 134,168 bp in length and shared the highest similarity (90% coverage, 99% identity) with pELF1 (accession number: LC495616) in GenBank (Supplementary Figure 3). The *vanA* gene cluster of SC1762 was located on a canonical Tn*1546*-like structure (Figure 7). However, the *vanH* gene in the mutant (pSC1762-D-*vanA*) was incomplete and truncated by an approximately 9 kb putative transposon (IS*1216E*-IS*Efa4-repB*-IS*Efa7*-IS*1216E*). The transposon was flanked by two copies of IS*1216E* in the same orientation. The sequences of IS*1216E*-IS*Efa4* and IS*Efa4-repB*-IS*Efa7*-IS*1216E* were identical to those of Part II and Part I of pSC1762-*vanA*, respectively, suggesting a rearrangement of the IS-associated region (Figure 7). Furthermore, a 7-bp direct repeat (CTTTGGC), identical to the sequence (positions 75 to 81) of the *vanH* gene, was identified around the transposon. The results indicated a transposition process in the *vanA* gene cluster mediated by an IS*1216E-*based composite transposon.

### Fitness Costs in *Enterococcus faecium* After the Acquisition of Daptomycin Resistance

**FIGURE 8:**
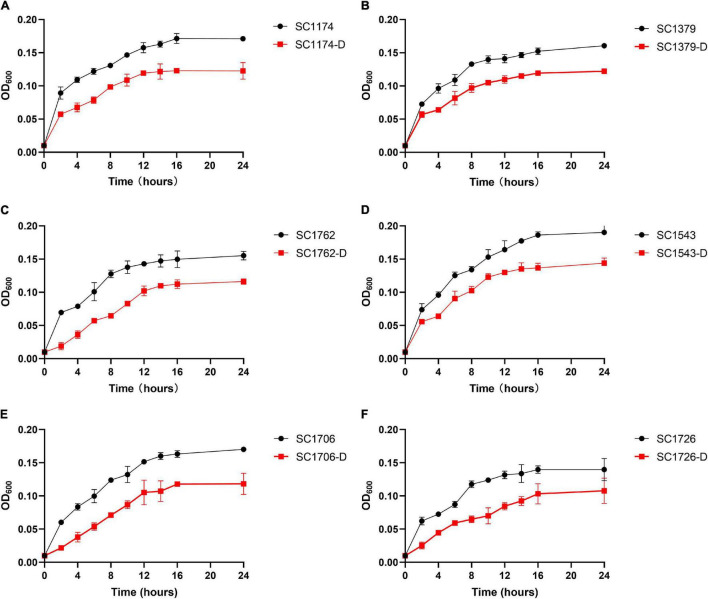
Bacterial growth curves for the parental strains and evolved strains. **(A–F)** show the growth curves for SC1174, SC1379, SC1762, SC1543, SC1706, SC1726, and their corresponding evolved DAP-R mutants, respectively. The standard deviation of three independent experiments is indicated in error bars. One-way analysis of variance (ANOVA) was used in statistical analysis (*P* < 0.05 vs. respective DAP-S parental strains).

**FIGURE 9:**
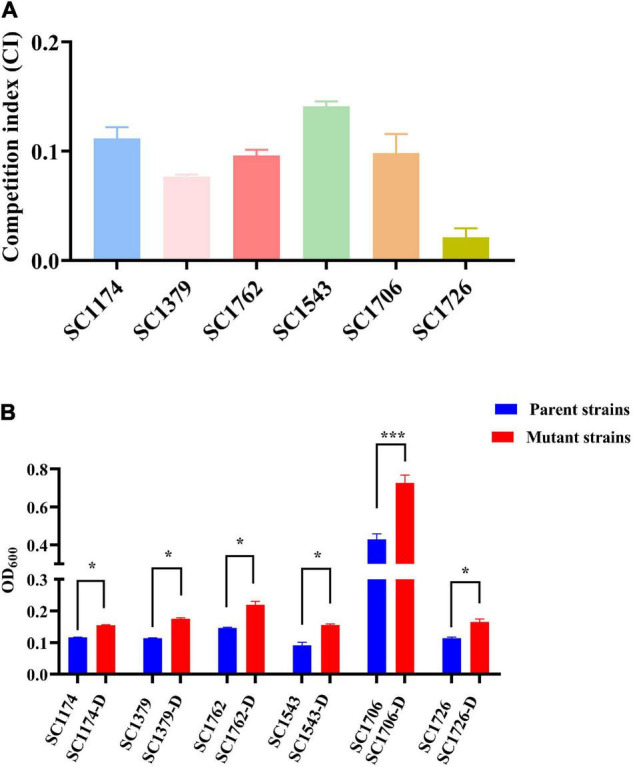
*In vitro* competition index and biofilm formation ability. **(A)** The *in vitro CI* in parental isolates and mutants. **(B)** Levels of biofilm formation in parental isolates and mutants. -D, evolved DAP-R mutants. The error bars show standard deviation from three independent experiments, and the absorbance value at *OD*_600_ was used to compare the biofilm formation ability of the DAP-S parental strains and respective evolved DAP-R strains with paired Student’s *t*-test (*P < 0.05 and ***P < 0.001).

**FIGURE 10:**
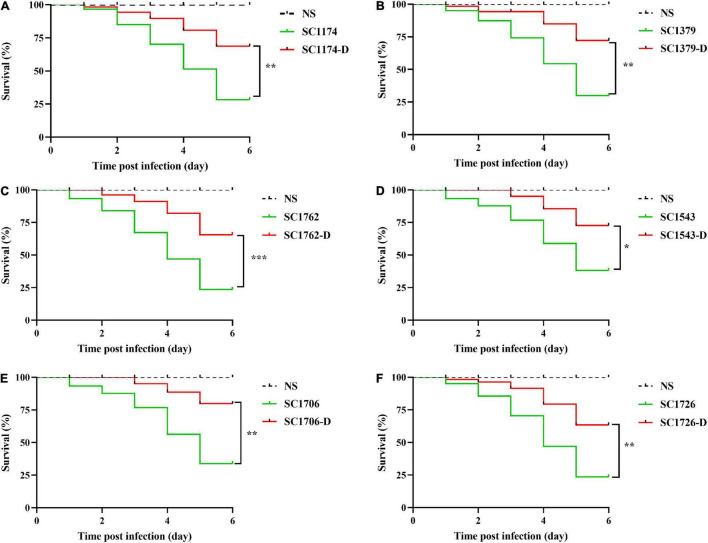
Infection of *Galleria mellonella* larvae. **(A–F)** show the survival curves of *Galleria mellonella* larvae infected with SC1174, SC1379, SC1762, SC1543, SC1706, SC1726, and their corresponding evolved DAP-R mutants, respectively. Survival data are plotted using the Kaplan–Meier method and expressed as percentage of survival vs. time. The corresponding *P*-values are given in the parenthesis for each group using the log-rank test (*P < 0.05; **P < 0.01; and ***P < 0.001).

To test the fitness difference between DAP-R mutants and their susceptible counterparts, we performed growth rate, *in vitro* competition, biofilm, and *G. mellonella* infection assays. The growth rate and bacterial density of the DAP-R mutants were lower than those of the original strains (Figure 8). Results of competition experiments indicated that DAP-R mutants showed a marked decrease in fitness (Figure 9A). Moreover, the biofilm-forming ability of evolved DAP-R strains was significantly higher than that of the corresponding parental strains; this difference was especially stark for SC1706-D (Figure 9B). To evaluate the virulence changes in the evolved strains, an infection model was generated using *G. mellonella* larvae. At 6 days post infection, the mortality of larvae infected with the DAP-R mutants was lower than that of larvae infected with the corresponding DAP-S strains, and no mortality was observed in the control injected with NS (Figure 10).

## Discussion

With the extensive, continuous, and inappropriate use of antibiotics, bacterial resistance to antimicrobial agents has become increasingly common, posing a serious threat to public health around the world (Watkins and Bonomo, 2016). In this context, CS—a beneficial trade-off that allows the reversal of antibiotic resistance—has attracted the attention of researchers globally (Barbosa et al., 2017). A better understanding of the evolutionary processes related to antibiotic resistance and the molecular mechanisms of CS could potentially inform effective therapeutic strategies in the future. However, the evolution of DAP resistance and acquired CS are still poorly understood. In this study, we investigated the evolution and mechanisms of DAP resistance, and further explored the possible mechanisms underlying the reversal of vancomycin and teicoplanin resistance in the evolved DAP-R strain SC1762-D.

In this study, six wild-type strains rapidly and successfully developed stable DAP resistance under DAP exposure. This indicated that under clinical conditions, DAP doses and regimens should be controlled strictly while treating bacterial infections, even though DAP is a useful treatment option for patients with persistent infections. Laboratory evolution often leads to differences in physiological characteristics between adapted strains and parental strains, which are usually related to a fitness cost (Kang et al., 2017; Li et al., 2017). In our study, we identified reduced growth, *in vitro* competition, and virulence among the evolved DAP-R isolates, although biofilm formation increased and was associated with the acquisition of DAP resistance (Guo et al., 2012; Saffari et al., 2017).

Genetic alterations were analyzed to gain insights into the evolution and mechanisms of DAP resistance. Consistent with previous studies, mutations in *liaFSR*, *yycFG*, and *cls* were detected in evolved DAP-R *E. faecium* strains (Arias et al., 2011; Munita et al., 2012). As mentioned previously, *cfa* mutations are associated with DAP resistance; however, no mutation in *cfa* was detected in the evolved DAP-R isolates in this study. This may be due to differences in genetic heritability and backgrounds between strains. Moreover, the *cfa* mutation could be more likely involved in spontaneous resistance to DAP than in laboratory-evolved resistance. In our study, we observed that the mutation sites in DAP-R genes differed among evolved DAP-R mutants, illustrating the diversity and dynamicity of DAP-R evolution pathways in *E. faecium*. We also compared the relationship between genetic mutation sites and different DAP-R levels. As shown in Figure 2, the evolved DAP-R resistance strain SC1706-D with the highest resistance level (512 μg/ml) only had few mutation sites in two genes, which was less than other evolved DAP-R strains. Additionally, these mutation sites detected in SC1706-D also appeared in SC1174-D and SC1762-D. These data revealed that there was no significant correlation between genetic mutation sites and different DAP-R levels. In fact, except for genetic mutations, changes in the positive charge on the cell surface and the thickness of the cell wall also contributed to the evolution of DAP resistance, as mentioned earlier. In other words, the different sites of gene mutation may not be tightly related to the DAP resistant levels. Moreover, we detected a dramatic increase in the surface positive charge and cell-wall thickness in evolved DAP-R mutants compared with the parental strains, along with upregulation of *dltABCD* and/or *tagGH*. These findings are consistent with the results reported in previous studies (Mishra et al., 2012; Bayer et al., 2016; Miller et al., 2016).

Cross-resistance and collateral sensitivity are common evolutionary trade-offs during adaptive bacterial evolution (Lozano-Huntelman et al., 2020). The most clinically important trade-off for DAP resistance in the *E. faecium* strain SC1762-D was perhaps the resensitization to vancomycin and teicoplanin. In *E. faecium*, active efflux confers bacteria with the ability to counteract a wide range of antimicrobials, including glycopeptides (Nishioka et al., 2009). Azimi and Rastegar Lari (2017) reported that CS between aminoglycosides and beta-lactam antibiotics depends on active proton pumps. Thus, we hypothesized that DAP induction may inhibit efflux pump activity, preventing the pumping of intracellular glycopeptides out of the cell and restoring the sensitivity of SC1762-D to glycopeptides. Contrary to our expectations, there was no difference in the efflux pump phenotype between the CS group and non-CS group, indicating that efflux pump activity may not be involved in the CS between DAP and glycopeptides.

Further, we tested the hypothesis that mutations or expression-level changes in the *vanA* gene cluster drove the development of CS in SC1762-D. No deletion or mutation was observed in the *vanA* gene cluster in the mutant strains. Interestingly, in the CS group, the expression of the *vanA* gene cluster (except *vanX*) was significantly lower in SC1762-D than in the corresponding parental strain. However, in the non-CS group, i.e., SC1174-D, hyperexpression of this gene cluster was detected. These results suggested that the reduced expression of *vanA* gene cluster was mainly responsible for CS. In addition, we also tried to analyze the changes in the intergenic regions of the pSC1762-*vanA* and pSC1762-D-*vanA* plasmids, such as promoter activity, to explain the differences in *vanA* gene cluster expression. But no differences in promoter level or other differences (such as gene mutations and sequence deletions) were observed between the *vanA* gene clusters of SC1762 and SC1762-D, except for the insertion of IS elements.

A previous study reported that mobile genetic elements, such as IS elements, decrease or silence the expression of resistance-related genes (Sun et al., 2019). In this study, an IS*1216E-*based composite transposon was formed in the DAP-R mutant SC1762-D and disrupted the *vanH* gene, likely affecting the structure and expression of the *vanA* gene cluster. Vandecraen et al. (2017) confirmed that the insertion or transposition of IS elements is affected by pathogens and environmental signals, offering an adaptive strategy for bacteria and promoting genetic variability against environmental challenges. We found that IS insertion may be an adaptive strategy used by bacteria to survive under the selection pressure of DAP. However, unlike other *vanA* genes, *vanX* did not show decreased expression in SC1762-D. A possible explanation is that the function or activity of *vanX* was not influenced by the IS insertion (Monteiro da Silva et al., 2020).

The pSC1762-*vanA* plasmids carrying the *vanA* gene cluster have a high homology with the novel mobile linear pELF1 plasmid detected by Japanese scholars (Hashimoto et al., 2019). Hence, these two plasmids could also have the mobility and transferability observed in pELF1. Recent studies have confirmed that the *vanA* gene cluster can be disseminated clonally but also horizontally by plasmid dissemination or Tn*1546* transposition between different genomic locations (Freitas et al., 2013; Lanza et al., 2015; Freitas et al., 2016; Arredondo-Alonso et al., 2021). Hence, it must be noted that pSC1762-*vanA* plasmids may confer the risk of antibiotic resistance transmission, and this should be monitored carefully.

Our research also has some limitations. Although mobile ISs were detected in both pSC1762-*vanA* and pSC1762-D-*vanA* plasmids, why IS insertion occurs in the *vanA* gene cluster and not in other resistance genes remains unclear. Moreover, it is not known whether the CS between DAP and glycopeptides is universal. In the future, we will further explore these questions in a focused manner.

## Conclusion

In short, the findings of this investigation provide convincing evidence that *E. faecium* can easily acquire high-level DAP resistance *in vitro*. This resistance can be attributed to genetic mutations and changes in cell-wall thickness and cell-membrane charge. Further, this is the first study reporting CS between DAP and glycopeptides, which is related to the decreased expression of the *vanA* gene cluster owing to IS insertion. These results provide a proof-of-concept and support the sequential and combinational use of DAP with glycopeptides for the treatment of VRE infections.

## Data Availability Statement

The datasets presented in this study can be found in online repositories. The names of the repository/repositories and accession number(s) can be found in the article/Supplementary Material.

## Author Contributions

WZ conducted the experiments, analyzed the data, and wrote the manuscript. LF, CQ, and TC participated in experiments. SW, YZ, and XZ took part in analysis of results. LW and SL participated in the analysis of results. TZ and YS helped to design the study. All authors contributed to the article and approved the submitted version.

## Conflict of Interest

The authors declare that the research was conducted in the absence of any commercial or financial relationships that could be construed as a potential conflict of interest.

## Publisher’s Note

All claims expressed in this article are solely those of the authors and do not necessarily represent those of their affiliated organizations, or those of the publisher, the editors and the reviewers. Any product that may be evaluated in this article, or claim that may be made by its manufacturer, is not guaranteed or endorsed by the publisher.

## Footnotes

1
http://www.ncbi.nlm.nih.gov/BLAST

2
https://inkscape.org/en/
