# Supplementary material for: Acquisition of Daptomycin Resistance by Enterococcus faecium Confers Collateral Sensitivity to Glycopeptides
Source: Front Microbiol. 2022 Apr 12;13:815600. doi: 10.3389/fmicb.2022.815600 (PMC9041417; doi:10.3389/fmicb.2022.815600)
Supplement: Supplementary file 1 [file Data_Sheet_1.doc]

Supplementary Material

# 1 Supplementary Table

## Table S1 Primers were used in this study

| Primers | Sequences (5*'*→3*'*) |
| --- | --- |
| Primers for PCR |  |
| *liaF*-F | CATCTAAAGTCCTATAGCCAAC |
| *liaF*-R | CAGCTAGCATCGCTCTGGAT |
| *liaS*-F | TACCTTTGGGCGTAGCCATCCGA |
| *liaS*-R | CCGGTCGAAGTGTCATCGCC |
| *liaR*-F | CCAGTCTGAGATGCGTGCGC |
| *liaR*-R | GCCACAACAGCATGAGTGGCT |
| *yycF*-F | GGGGATGCGAAAGAAGAAGCA |
| *yycF*-R | AAGAGCCCCGATAACGGCAT |
| *yycG*-F | AGAAGAGGCAGCACCTGCGGA |
| *yycG*-R | AAGGCGCATTATAGGCCAACTCAAA |
| *cls*-F | TATTCACCTTGGTAGGCTTC |
| *cls*-R | TTTTTGTCACAGTCCCTTTAATCTT |
| *cfa*-F | GCCAACTCAAGTGTTTGCGG |
| *cfa*-R | AAGAGCGCTGTTCGCTACTG |
| *vanA*-F | GGAGACAGGAGCATGAATAGAATA |
| *vanA*-R | CGTCCCAACGAACACCGTGTACTA |
| *vanH*-F | ATGAATAACATCGGCATTACTGT |
| *vanH*-R | GATACGCATGCTCCTTGAGCAAC |
| *vanR*-F | TGTGAAAGGGTGATAACTATGAG |
| *vanR*-R | GCTACCACAACAATTGCAACGATA |
| *vanS*-F | GTTAGCTCCGAGCTGCTATTTCAT |
| *vanS*-R | AGTCTACCGTGTAAGAACGAGCCG |
| *vanX*-F | CGCTTGATCGTATTAGCGTTAAAG |
| *vanX*-R | GGGTTTCCTGCCGAAAGAGTTAGC |
| *vanY*-F | CTTCCAACTATATAGTTAAGGAGG |
| *vanY*-R | TTCGTTGGTTTCATTCCGCCATCC |
| *vanZ*-F | CCCACGTTAACTAGGACATTGCTA |
| *vanZ*-R | TCACTTCACACCTACGGGCGAGTT |
| Primers for qRT-PCR |  |
| *dltA*-F | tcgattcccaaatgcacgga |
| *dltA*-R | gccaatcggtagtcggtcat |
| *dltB*-F | tcgttggcatatgacgctgt |
| *dltB*-R | accacgtcaatccatgccat |
| *dltC*-F | ctgaattcgagcgagaggattg |
| *dltC*-R | caacccttcaatgcagttgc |
| *dltD*-F | tttcggcccggttgttttag |
| *dltD*-R | tcgcatctcctttcaacacg |
| *tagG*-F | tggtgttggactgaaagcac |
| *tagG*-R | tttcgtcgtgggcaagatac |
| *tagH*-F | acgtgacgaaagcggaattg |
| *tagH*-R | actgttcgtagcgcaattcc |
| *vanR*-F | TATAACGAAGCCCTTTCGCC |
| *vanR*-R | TTAATGACAAGGCCGGAGTG |
| *vanS*-F | GGACTCTGGAAAAGCGAGAG |
| *vanS*-R | GGGCGTTTTAATATCGTGCG |
| *vanH*-F | GACATGACAGTTGGTGTGGT |
| *vanH*-R | CTCTATACTTCGGCTGCGAC |
| *vanA*-F | CCGGTATCCCTTTTGTAGGC |
| *vanA*-R | GCTATCCCAGCATTTTTCGC |
| *vanX*-F | CAAAATCAAGCCATAGCCGC |
| *vanX*-R | CTCCCCATTGGTACAAGCTC |
| *vanY*-F | TGTAAAGGGTGGCGTTAGTC |
| *vanY*-R | CCTGCTGGTAAGGCATACTC |
| *vanZ*-F | TGGAGGCTTTCTTGGACTGA |
| *vanZ*-R | GGGTACGGTAAACGAGCAA |
| *16S rRNA*-F | AGGGGATAACACTTGGAAACA |
| *16S rRNA*-R | TTCGCGACTCGTTGTACTTC |

## Table S2 Genome features of SC1762 and its mutant

| **Strain** | **Sequence ID** | **Sequence Length (bp)** | **GC Content (%)** | **Sequence Type** | **ARGs** |
| --- | --- | --- | --- | --- | --- |
| **SC1762** | chr | 2,814,835 | 38.19 | circular | *aac(6')-Ii*, *msr(C)* |
|  | Plasmid 1 | 224,638 | 35.61 | circular | *aac(6')-aph(2'')* |
|  | pSC1762-vanA | 134,168 | 33.99% | linear | *aph(3')-III*, *erm(B)*, *vanA-cluster* |
|  | Plasmid 2 | 63,475 | 32.06 | circular | - |
|  | Plasmid 3 | 23,182 | 34.79 | circular | - |
|  | Plasmid 4 | 7,838 | 33.48 | circular | - |
|  | Plasmid 5 | 6,326 | 35.55 | circular | - |
|  | Plasmid 6 | 6,175 | 35.47 | circular | - |
|  | Plasmid 7 | 5,275 | 41.14 | circular | - |
|  | Plasmid 8 | 4,463 | 31.95 | circular | - |
|  | Plasmid 9 | 2,056 | 37.79 | circular | - |
|  | Plasmid 10 | 1,979 | 38.91 | circular | - |
| SC1762-D | chr | 2,816,191 | 38.19 | circular | *aac(6')-Ii*, *msr(C)* |
|  | Plasmid 1 | 224,638 | 35.61 | circular | *aac(6')-aph(2'')* |
|  | pSC1762-D-vanA | 134,985 | 34.00% | linear | *aph(3')-III*, *erm(B)*, *ΔvanA-cluster* |
|  | Plasmid 2 | 63,475 | 32.06 | circular | - |
|  | Plasmid 3 | 23,182 | 34.79 | circular | - |
|  | Plasmid 4 | 7,838 | 33.48 | circular | - |
|  | Plasmid 5 | 6,326 | 35.55 | circular | - |
|  | Plasmid 6 | 6,175 | 35.47 | circular | - |
|  | Plasmid 7 | 5,275 | 41.14 | circular | - |
|  | Plasmid 8 | 4,463 | 31.95 | circular | - |
|  | Plasmid 9 | 2,056 | 37.79 | circular | - |
|  | Plasmid 10 | 1,979 | 38.91 | circular | - |

# 2 Supplementary Figure

# 2.1 Figure S1 Adaptive laboratory evolution of *E. faecium* isolates


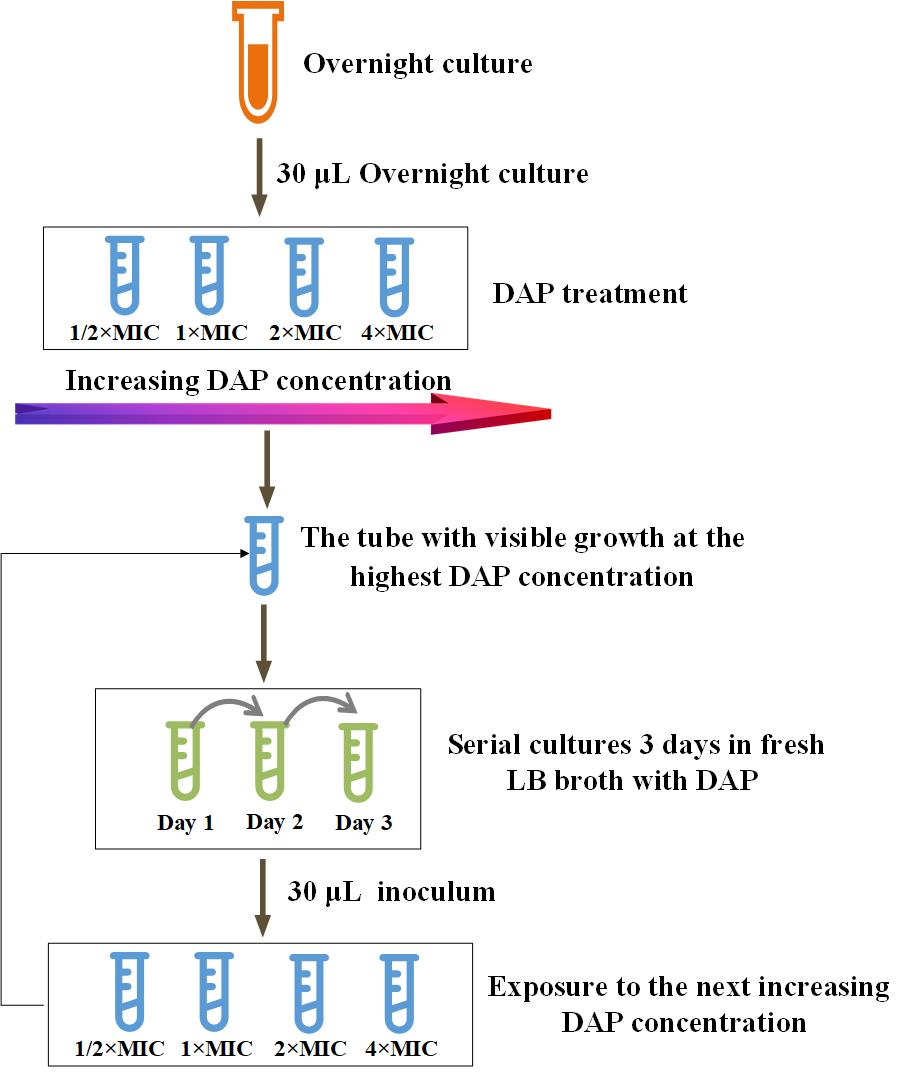


**Figure S1 Adaptive laboratory evolution** **of *E. faecium* isolates**

# 2.2 Figure S2 Gel electrophoresis of PCR products


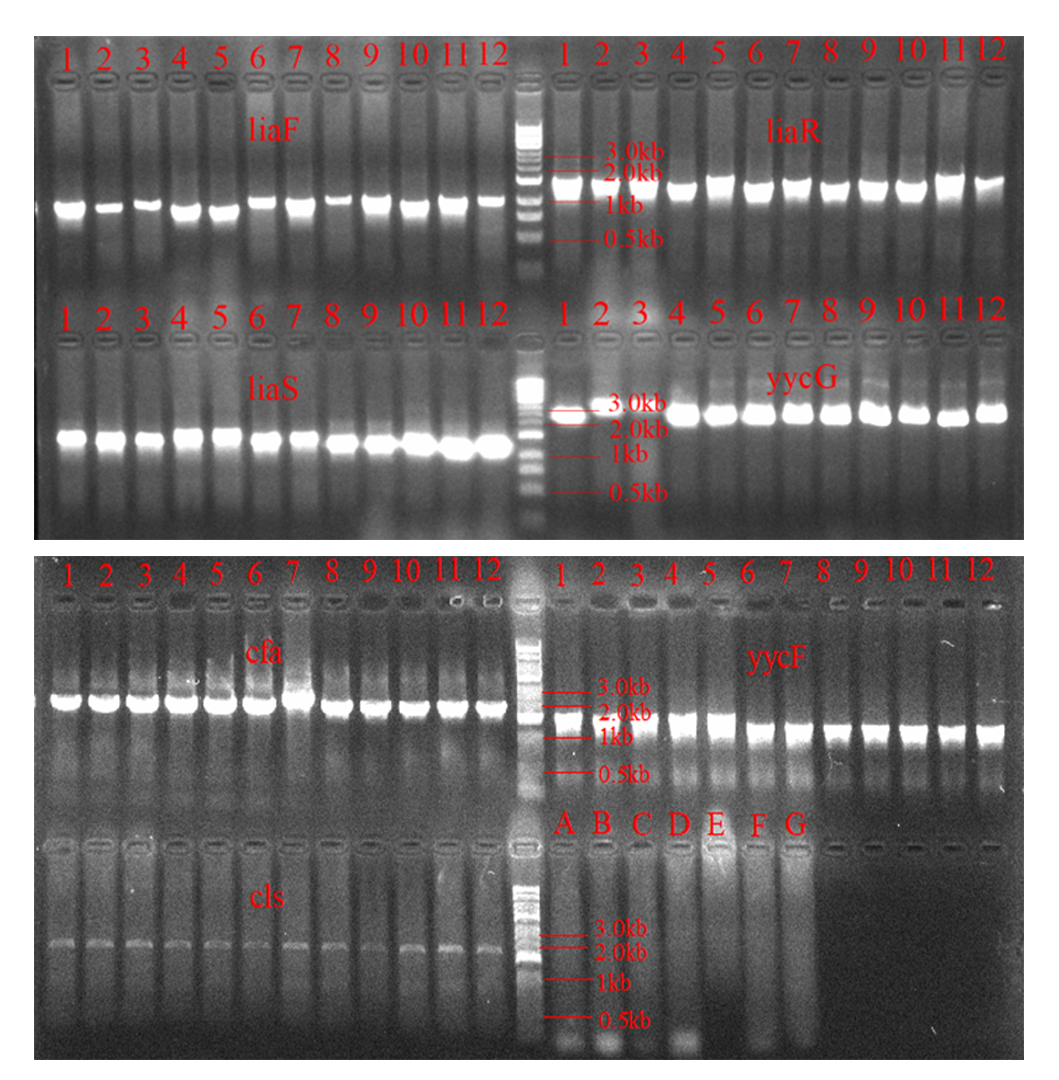


**Figure S2** **Gel electrophoresis of PCR products.** Lines 1-12: SC1174, SC1174-D, SC1379, SC1379-D, SC1762, SC1762-D, SC1543, SC1543-D, SC1706, SC1706-D, SC1726, and SC1726-D, respectively; A-G: Negative controls for *liaF*, *liaR*, *liaS*, *yycG*, *cfa*, *yycF*, and *cls*, respectively.

# 2.3 Figure S3 Genomic comparison of the pSC1762-*vanA* plasmid with pELF1 plasmid


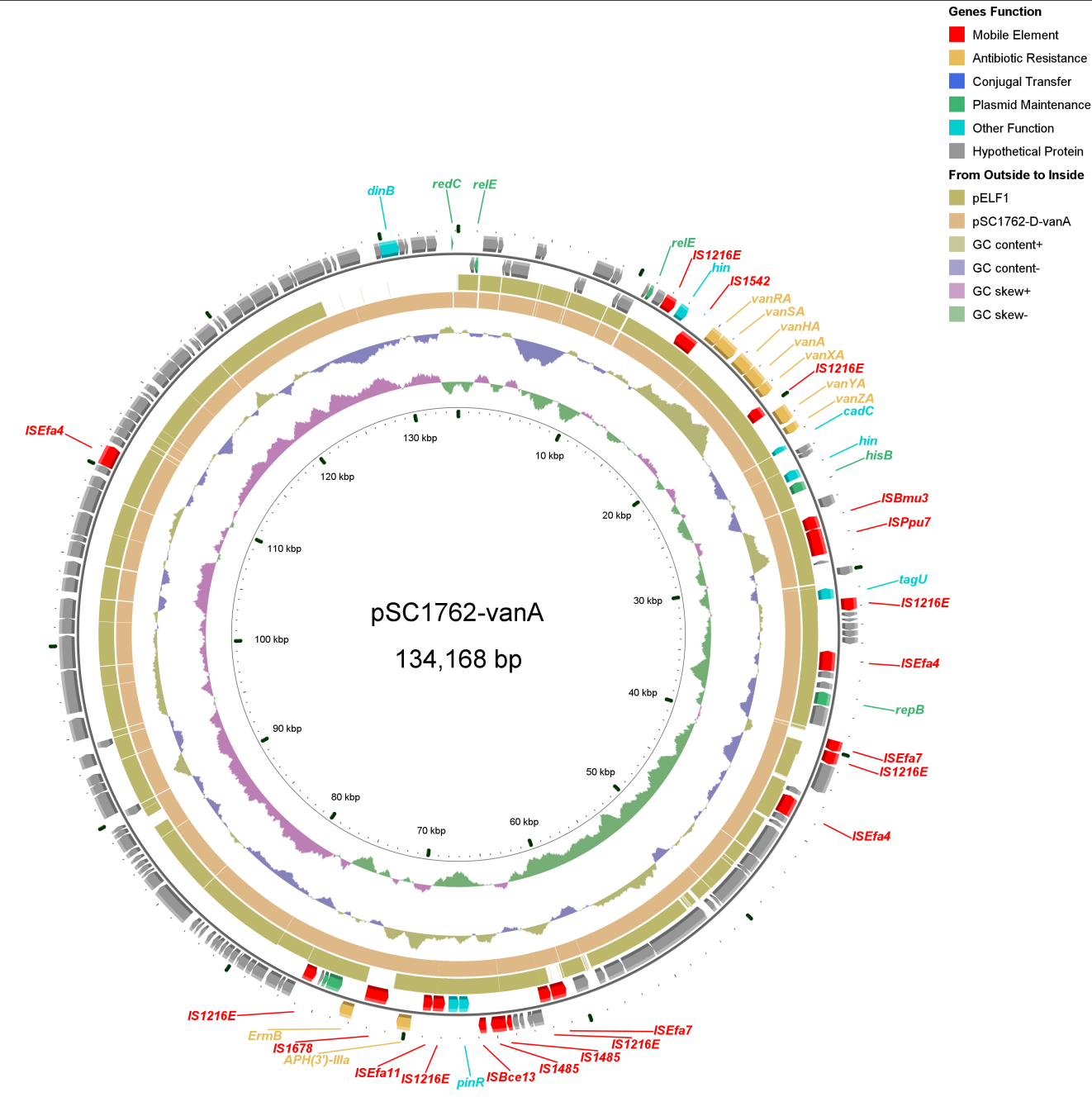


**Figure S3** **Genomic comparison of the pSC1762-*vanA* plasmid with** **pELF1 plasmid.** Genes are denoted by arrows and are colored based on the gene functional classification.
